# High frequency of the *TARDBP* p.M337 V mutation among south-eastern Chinese patients with familial amyotrophic lateral sclerosis

**DOI:** 10.1186/s12883-018-1028-1

**Published:** 2018-04-05

**Authors:** Guo-rong Xu, Wei Hu, Ling-Ling Zhan, Chong Wang, Liu-Qing Xu, Min-Ting Lin, Wan-Jin Chen, Ning Wang, Qi-Jie Zhang

**Affiliations:** 1 Department of Neurology and Institute of Neurology, First Affiliated Hospital, Fujian Medical University, 20 Chazhong Road, Fuzhou, 350005 China; 2 Fujian Key Laboratory of Molecular Neurology, Fuzhou, China

**Keywords:** Amyotrophic lateral sclerosis, Familial, *TARDBP*, Genotype-phenotype analysis

## Abstract

**Background:**

Amyotrophic lateral sclerosis (ALS) is a devastating motor neuron disease characterized by substantial clinical and genetic heterogeneity. Thus far, only a few *TARDBP*-ALS families have been reported in China, and no mutation analysis has been reported in south-eastern China.

**Methods:**

Seven index cases from ALS families negative for *SOD1* and *FUS* mutations were screened by Sanger sequencing for *TARDBP* gene exons 2-6. *TARDBP* exon 6 was analysed in 215 sporadic ALS patients.

**Results:**

Two *TARDBP* mutations in exon 6 (p.M337 V and p.G348C) were identified in 5 unrelated families. Four of these 5 families carried the same p.M337 V mutation (family 1II3, family 2II6, family 3II4, and family 4II4), and the p.G348C mutation was identified in family 5 (II5). Among the 215 sporadic patients, only a single nucleotide polymorphism (p.A366A) was detected in 5 patients, and no responsible mutation was identified. Among the *TARDBP-*linked familial ALS patients, the average age of onset was 57.0 ± 4.7 years, and a trend towards higher rates of bulbar (50.0%, 6/12) onset and upper limb (41.7%, 5/12) onset than lower rates of limb onset (8.3%, 1/12) was observed. Furthermore, ALS patients with *TARDBP* mutations showed a benign disease course, and the average survival was 106.5 ± 41.8 months (*n* = 8).

**Conclusions:**

We found a high frequency of the *TARDBP* p.M337 V mutation in familial ALS in south-eastern China. The *TARDBP-*linked ALS patients showed a benign disease course and prolonged survival.

**Electronic supplementary material:**

The online version of this article (10.1186/s12883-018-1028-1) contains supplementary material, which is available to authorized users.

## Background

Amyotrophic lateral sclerosis (ALS) is a typical form of motor neuron disease characterized by selective degeneration of both the upper motor neurons (UMNs) and the lower motor neurons (LMNs) in the cerebral cortex, brain stem, and spinal cord, with an incidence of approximately 1-2 per 100,000 people [1]. Typically, ALS patients initially present with symptoms in the upper limbs later in life (40-60 years old). The disease then spreads to the trunk and bulbar muscles, and patients ultimately die from respiratory failure, with a mean survival of 3-5 years [2]. ALS is a progressive and incurable disease with no effective treatment available.

Currently, the complete aetiology of ALS is unclear. Approximately 5-10% of ALS patients have a family history of ALS (defined as familial ALS), and the remainder of patients have sporadic ALS. ALS is a neurodegenerative disorder with substantial genetic heterogeneity, and more than 30 genes are associated with familial ALS. In the Caucasian population, chromosome 9 open reading frame 72 (*C9ORF72*, MIM: 614260) repeat expansions are the most common genetic cause of ALS; in China, Cu/Zn superoxide dismutase 1 (*SOD1,* MIM: 147450), fused in sarcoma/translated in liposarcoma (*FUS/TLS,* MIM: 137070), and TAR DNA-binding protein (*TARDBP,* MIM: 605078) are the main causative genes among ALS patients [2, 3]. However, the frequencies of gene mutations are diverse between different populations, and this diversity has been linked to different genetic backgrounds. Thus far, only a few *TARDBP*-ALS families have been reported in China, and no mutation analysis has been reported in south-eastern China [4–7].

In this study, 7 *SOD1*- and *FUS*-negative ALS families and 215 sporadic ALS patients were enrolled to screen the *TARDBP* mutation. Two *TARDBP* mutations in exon 6 (c.1009A > G, p.M337 V and c.1042G > T, p.G348 C) were identified in 5 families (family 1II3, family 2II6, family 3II4, family 4II4, and family 5II5). Interestingly, 4 unrelated ALS families (families 1-4) carried the same p.M337 V mutation. No disease-causing mutations were identified in the sporadic ALS patients. *TARDBP*-linked familial ALS patients often showed upper limb and bulbar onset and had a benign disease course.

## Methods

### Patients

Seven unrelated *SOD1*- and *FUS*-negative ALS families, including 13 ALS patients, were enrolled. All the families were of the Han ethnic group, were recruited from different regions of south-eastern China, and had at least one affected family member. In addition, 215 sporadic ALS patients, including 148 males and 67 females, were enrolled in this study. The average age at onset for sporadic ALS was 54 ± 11.5 years. All the patients were diagnosed with possible, probable, or definite ALS according to the revised EI Escorial criteria [8]. The patients were recruited from the Department of Neurology, First Affiliated Hospital of Fujian Medical University from August 2008 to August 2016. Clinical profiles, including the age at onset, site at onset, disease course, laboratory data, electromyography (EMG) results, motor function, and pulmonary function, were analysed.

### Gene mutation analysis

Genomic DNA was extracted from peripheral blood using a Genomic Extraction Kit (Qiagen, Germany). First, the survival of motor neuron gene (*SMN1* and *SMN2,* MIM: 600354) mutations were screened to exclude the possibility of spinal muscular atrophy (SMA). For the male patients, CAG expansion in the androgen receptor gene (*AR,* MIM: 313700) was further detected to exclude spinal and bulbar muscular atrophy (SBMA). The patients negative for mutations in the *SMN* and *AR* genes were then selected for ALS gene screening. *SOD1*- and *FUS*-negative familial ALS patients were selected to screen for *TARDBP* gene mutations by Sanger sequencing, and the primer sequences are listed in Additional file 1: Table S1.

## Results

### Gene mutation analysis of the ALS patient cohort

**Fig. 1:**
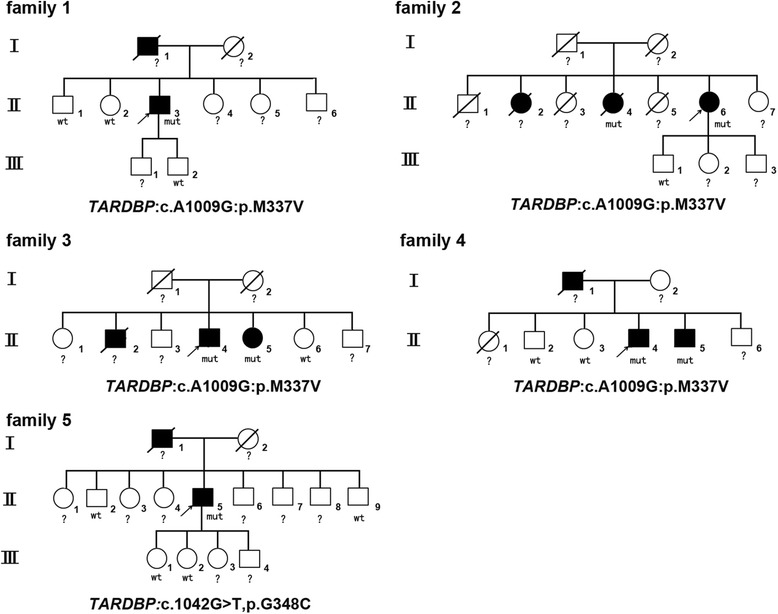
Pedigrees and gene mutations of the 5 ALS families. The squares represent males. The circles represent females. The black symbols represent affected patients. The arrows indicate the probands. The diagonal lines across symbols represent deceased patients. “mut” indicates the affected carriers of the gene mutation. “wt” indicates healthy family members without the gene mutation. “?” indicates an undetected gene mutation due to subject death or refusal to participate in further genetic analysis

Among the 7 unrelated *SOD1*- and *FUS*-negative ALS families, we identified 2 known *TARDBP* mutations (*TARDBP*: c.1009A > G, p.M337 V and *TARDBP*: c.1042G > T, p.G348C) in 5 families. Interestingly, the *TARDBP*: c.1009A > G, p.M337 V mutation was found in family 1, family 2, family 3, and family 4 (Fig. 1). Among the 215 sporadic patients, 5 patients carried a heterozygous variant (c.1098C > G, p.A366A), which was confirmed to be a single nucleotide polymorphism. However, no responsible gene mutation was identified in exon 6 of the *TARDBP* gene for this cohort of sporadic patients.

### Clinical features of 4 pedigrees with the *TARDBP* c.1009A > G, p.M337 V mutation

Beginning at 58 years of age, the proband of family 1 (II3) developed progressive speech and swallowing difficulties. Initially, he was diagnosed with vocal cord polyps in the ear-nose-throat department and underwent vocal cord polypectomy. However, his symptoms did not resolve and gradually worsened. Muscular weakness and atrophy spread to his upper limbs and then to his lower limbs. Neurological exam indicated mild muscle atrophy in his tongue and limbs with increased tendon reflexes and the Chaddock sign bilaterally. EMG testing revealed widespread neurogenic lesions. He died from respiratory failure 9 years later. His father (I1) also suffered from similar symptoms and died 6 years after symptom onset.

The proband of family 2 (II6) was a 61-year-old female who suffered from right upper limb stiffness and weakness for 2 years. One year later, she developed swallowing and speaking difficulties. Neurological exam showed dysarthria and choking. Muscle weakness was found in the neck flexor muscle (grade 4) and right limbs (grade 5-) without significant muscle atrophy. The jaw reflex and Babinski sign were positive. EMG revealed widespread neurogenic lesions in the sternocleidomastoid and the cervical, lumbar, and thoracic paraspinal muscles (T8). Her older sister (II2) also developed dysarthria at 60 years of age and died 12 years later. Another sister (II6) showed speaking and swallowing difficulties beginning at 58 years of age. Her symptoms progressed, and limb muscle weakness and atrophy were noted after 1 year. EMG testing also revealed widespread neurogenic lesions. She was diagnosed with bulbar-onset ALS and died from respiratory failure after surviving for 120 months.

The proband of family 3 (II4) was a male patient who developed muscle weakness in the right upper limb at the age of 62 years that spread to the left upper limb and bulbar muscles 2 years later. Neurological exam showed significant muscle atrophy in the bilateral hand muscles, including the interosseous and thenar muscles. Mild muscle weakness (grade 5-) was detected in the limbs with increased tendon reflexes bilaterally. Neurogenic lesions were observed in the muscles innervated by the cervical, lumbar, and thoracic (T8, T9, and T10) spinal cord. His sister (II5) was also admitted to our department and presented with muscle weakness in her lower left limb 8 months prior to admission and developed speaking difficulties and choking 2 months later. Neurological exam showed mild muscle weakness (grade 5-) in the neck and lower limb muscles without muscle weakness. Hypermyotonia was observed in the upper and lower left limbs. Tendon reflexes were significantly increased in all the limbs. Additionally, the Hoffmann and Babinski signs were positive. EMG testing revealed widespread neurogenic lesions. At the age of 50 years, his older brother (II2) suffered from muscle weakness in the upper limbs that spread to his bulbar muscles. He was also diagnosed with ALS and died due to respiratory failure 3 years later.

The proband of family 4 (II4) was a 59-year-old male who developed dysarthria at 54 years of age. In the years following symptom onset, his symptoms slowly progressed, and the affected limb muscles caused walking and writing abnormalities. Neurological exam showed muscular fasciculation without significant muscle atrophy. The tendon reflexes were increased in all the limbs. The Chaddock sign and palm-chin reflex were positive bilaterally. EMG testing revealed widespread neurogenic lesions. His father (I1) developed dysarthria at the age of 62 years and died 14 years later. His younger brother (II5) also carried the *TARDBP* c.1009A > G, p.M337 V mutation; however, he presented with no noticeable symptoms until recently.

### Clinical features of the pedigree with the *TARDBP* c.1042G > T, p.G348C mutation

The proband of family 5 (II5) was a 55-year-old male who first presented with muscle weakness and atrophy in his right hand at 50 years old. Two years later, his symptoms progressed to the left side, and he reported difficulty in raising his hands. Three years later, weakness and atrophy also developed in his lower limbs. Currently, he cannot walk unassisted. Neurological exam showed significant muscle weakness (grade 2-3) and atrophy in the upper and lower limbs without UMN signs. EMG revealed widespread neurogenic lesions. His father (I1) also presented with upper limb weakness at 50 years old and died 10 years later.

## Discussion

In this study, among the 7 unrelated *SOD1*- and *FUS*-negative ALS families, we detected 2 *TARDBP* mutations (c.1009A > G, p.M337 V and c.1042G > T, p.G348C) in 5 families. Importantly, 4 families carried the same *TARDBP* mutation, c.1009A > G, p.M337 V. Hou L et al. screened *SOD1*, *TARDBP*, and *FUS* gene mutations in ALS patients from central-southern China and revealed that *SOD1* (20%) and *FUS* (13.3%) mutations were the main causal mutations in familial ALS, but they did not detect any *TARDBP*-linked ALS families [9]. In a Brazilian research centre, Chadi G et al. reported that the most common gene mutations in familial ALS were *VAPB* (43.6%), *C9orf72* (12.8%), and *SOD1* (7.7%), whereas no *FUS* or *TARDBP* mutations were detected in any familial ALS subjects [10]. In Australia, McCann EP et al. also reported that the main gene mutations in familial ALS were *SOD1* (13.7%), *FUS* (2.4%), and *TARDBP* (1.9%) [11]. Our study indicated a high frequency of the *TARDBP* gene mutation in familial ALS in the south-eastern region of China.

Among the 5 ALS families with the *TARDBP* gene mutation in the present study, 12 symptomatic ALS patients and 1 asymptomatic patient were identified (Table 1). For the 12 symptomatic patients, including 8 males and 4 females, the average age of onset was 57.0 ± 4.7 years. In addition, the *TARDBP-*linked familial ALS patients presented a trend towards higher rates of bulbar (50.0%, 6/12) onset and upper limb (41.7%, 5/12) onset than lower rates of limb onset (8.3%, 1/12); this finding is consistent with a predominance of upper limb onset in *TARDBP*-linked ALS reported in a previous study [12]. Furthermore, similar to previous reports, ALS patients with *TARDBP* mutations showed a benign disease course and an average survival of 106.5 ± 41.8 months (*n* = 8) [13].

**Table 1:** Clinical features and gene mutation results of the 5 ALS families

family No.	patient	gender	age at onset(y)	site of onset	Clinical phenotype	EMG test	survival(m)	gene mutation
family 1	I1	male	60	bulbar	NA	NA	72	NA
	II3	male	58	bulbar	LMN + UMN	M,C,T,L	108	*TARDBP*:c.1009A > G,p.M337 V
family 2	II2	female	60	bulbar	NA	NA	144	NA
	II4	female	58	bulbar	LMN-dominant	M,C,T,L	120	*TARDBP*:c.1009A > G,p.M337 V
	II6	female	59	upper limb	LMN + UMN	M,C,T,L	> 52	*TARDBP*:c.1009A > G,p.M337 V
family 3	II2	male	50	upper limb	NA	NA	36	NA
	II4	male	62	upper limb	LMN + UMN	C,T,L	84	*TARDBP*:c.1009A > G,p.M337 V
	II5	female	61	lower limb	UMN-dominant	M,C,T,L	> 68	*TARDBP*:c.1009A > G,p.M337 V
family 4	I1	male	62	bulbar	NA	NA	168	NA
	II4	male	54	bulbar	LMN + UMN	M,C,T,L	> 64	*TARDBP*:c.1009A > G,p.M337 V
	II5	male	no symptoms					*TARDBP*:c.1009A > G,p.M337 V
family 5	I1	male	50	upper limb	NA	NA	120	NA
	II5	male	50	upper limb	LMN-dominant	M,C,T,L	> 59	*TARDBP*:c.1042G > T,p.G348C

*NA* not available, *LMN* lower motor neuron, *UMN* upper motor neuron, *EMG* electromyography, *M* medullary, *C* cervical, *T* thoracic, *L* lumbar

Furthermore, *TARDBP* p.M337 V ALS patients showed substantial clinical heterogeneity between and among families carrying the same mutation. These patients could exhibit a LMN-dominant presentation, an UMN-dominant presentation, or the typical presentation with both LMN and UMN involvement, which is similar to the cases that we reported in family 2 and family 3. In addition, in family 3, compared with affected members II4 and II5, II2 first showed symptoms at the age of 50 years and died from respiratory failure 36 months later, reflecting a relatively short disease duration, which may suggest intrafamilial clinical heterogeneity. We also found a p.M337 V carrier without any neurological symptoms in family 4, which may be explained by incomplete penetrance; no similar patient has been previously reported. In addition, Ju X et al. reported a pedigree with the p.M337 V mutation that showed cognitive impairment. In this study, none of the 4 families with the p.M337 V mutation developed cognitive impairment; this result is similar to the results from a Taiwanese cohort. The substantial clinical heterogeneity between p.M337 V mutation pedigrees indicated the potential presence of other underlying phenotype-modifying factors [14, 15]. Kühnlein P et al. described the first ALS patient with the p.G348C mutation who presented with early spinal onset (31 years) without cognitive impairment [16]. Del Bo R et al. also reported a 5-generation p.G348C mutation ALS family with 9 affected members, and the affected members showed highly variable clinical features, including the age at onset (36 to 67 years) and disease duration (36 to 60 months) [17]. In this study, p.G348C mutation ALS patients (family 5, I1 and II5) had an age of onset of 50 to 55 years and spinal onset, which are similar to the features of previously reported patients [16, 17]. Among different ethnic groups, due to different genetic backgrounds, patients with *TARDBP* gene mutations also show substantial clinical heterogeneity. According to Corcia P et al., approximately 51.3% of Caucasian ALS patients have upper limb onset, while 58.8% of Asian patients have bulbar onset [18]. Similarly, bulbar-onset patients accounted for 50% of the patients in this study.

In this study, only a single nucleotide polymorphism (c.1098C > G, p.A366A) was identified in 5 sporadic patients. However, no mutation was detected in exon 6 of the *TARDBP* gene in the 215 sporadic patients. Similarly, in southern China, Ye CH et al. detected no *TARDBP* exon 6 mutations in 207 sporadic ALS patients [19]. Huang R and Zou ZY demonstrated that the frequency of *TARDBP* gene mutations in Chinese sporadic ALS patients was approximately 0.61% to 0.73% [5, 20]. The c.1098C > G, p.A366A polymorphism, which may increase susceptibility to ALS, was also previously reported [5].

## Conclusions

*TARDBP* gene mutations are the common causal mutations in this cohort of familial ALS patients from south-eastern China, with a high frequency of the p.M337 V mutation. The *TARDBP-*linked ALS patients showed substantial clinical heterogeneity and a more benign disease phenotype with a longer disease duration.

## Additional file

Additional file 1: **Table S1.** The primer sequences for TARDBP gene. (DOC 19 kb)

## Abbreviations

ALS: Amyotrophic lateral sclerosis
*AR*: Androgen receptor gene
*C9ORF72*: Chromosome 9 open reading frame 72 repeat expansion
*FUS/TLS*: Fused in sarcoma/translated in liposarcoma
LMN: Lower motor neuron
SBMA: Spinal and bulbar muscular atrophy
SMA: Spinal muscular atrophy
*SMN*: Survival of motor neuron gene
*SOD1*: Cu/Zn superoxide dismutase 1
*TARDBP*: TAR DNA-binding protein
UMN: Upper motor neuron
