# Supplementary material for: High frequency of the TARDBP p.M337 V mutation among south-eastern Chinese patients with familial amyotrophic lateral sclerosis
Source: BMC Neurol. 2018 Apr 5;18:35. doi: 10.1186/s12883-018-1028-1 (PMC5887188; doi:10.1186/s12883-018-1028-1)
Supplement: Supplementary file 1 — Table S1. The primer sequences for TARDBP gene. (DOC 19 kb) [file 12883_2018_1028_MOESM1_ESM.doc]

Additional file 1: Table S1 The primer sequences for *TARDBP* gene

| exon 2 | F | CTGGAAGTCAGAACTCTGAC |
| --- | --- | --- |
|  | R | TCAGGAGACATTCTGCCACC |
| exon 3 | F | GCTTCTCATTTCTAGATGTAGG |
|  | R | AGAACCTAGGGAACATAGTG |
| exon 4 | F | TAAGCCACTGCATCCAGTTG |
|  | R | GATTTCATGAACACACCCTG |
| exon 5 | F | TGGTTCACTGCTATCCAAGG |
|  | R | AGGATGGTCTTGATCTGGTG |
| exon 6 | F | CATTGCTTATTTTTCCTCTGGC |
|  | R | TATACTCCACACTGAACAAACC |
